# Contemporary surgical practice in the management of anal fistula: results from an international survey

**DOI:** 10.1007/s10151-019-02051-5

**Published:** 2019-07-31

**Authors:** C. Ratto, U. Grossi, F. Litta, G. L. Di Tanna, A. Parello, V. De Simone, P. Tozer, D. DE Zimmerman, Y. Maeda

**Affiliations:** 1 Proctology Unit, Fondazione Policlinico Universitario A. Gemelli IRCCS, Largo A. Gemelli, 8, 00168 Rome, Italy; 2 Università Cattolica del Sacro Cuore, Rome, Italy; 3 National Bowel Research Centre, Queen Mary University of London, London, UK; 4 Statistics Division, The George Institute for Global Health, UNSW, Sydney, Australia; 5 Fistula Research Unit, St Mark’s Hospital and Academic Institute, London, UK; 6 Imperial College London, London, UK; 7 Department of Surgery, ETZ (Elisabeth-TweeSteden Hospital), Tilburg, The Netherlands; 8 Department of Colorectal Surgery, Western General Hospital, Edinburgh, UK

**Keywords:** Anal fistula, Survey, LIFT, VAAFT, Incontinence, Recurrence

## Abstract

**Background:**

Management of anal fistula (AF) remains challenging with many controversies. The purpose of this study was to explore current surgical practice in the management of AF with a focus on technical variations among surgeons.

**Methods:**

An online survey was conducted by inviting all surgeons and physicians on the membership directory of European Society of Coloproctology and American Society of Colon and Rectal Surgeons. An invitation was extended to others via social media. The survey had 74 questions exploring diagnostic and surgical techniques.

**Results:**

In March 2018, 3572 physicians on membership directory were invited to take part in the study 510 of whom (14%) responded to the survey. Of these respondents, 492 (96%) were surgeons. Respondents were mostly colorectal surgeons (84%) at consultant level (84%), age ≥ 40 years (64%), practicing in academic (53%) or teaching (30%) hospitals, from the USA (36%) and Europe (34%). About 80% considered fistulotomy as the gold standard treatment for simple fistulas. Endorectal advancement flap was performed using partial- (42%) or full-thickness (44%) flaps. Up to 38% of surgeons performed ligation of the intersphincteric fistula tract (LIFT) sometimes with technical variations. Geographic and demographic differences were found in both the diagnostic and therapeutic approaches to AF. Declared rates of recurrence and fecal incontinence with these techniques were variable and did not correlate with surgeons’ experience. Only 1–4% of surgeons were confident in performing the most novel sphincter-preserving techniques in patients with Crohn’s disease.

**Conclusions:**

Profound technical variations exist in surgical management of AF, making it difficult to reproduce and compare treatment outcomes among different centers.

**Electronic supplementary material:**

The online version of this article (10.1007/s10151-019-02051-5) contains supplementary material, which is available to authorized users.

## Introduction

Descriptions of anal fistula (AF) are found in the oldest known medical literature [1]. The reported incidence in four of the countries of the European Union ranges between 10.4 per 100,000 in Spain and 23.2 per 100,000 in Italy [2]. Common symptoms include pain, difficulty in sitting and discharge of pus/blood, and AF has a detrimental effect on quality of life that worsens in recurrent disease [3]. Although 90–95% of cases are classified as cryptoglandular in origin, AF is a common and disabling phenotype of Crohn’s disease (CD) [4]. Indeed, one-third of CD patients will develop an AF, with just one-third of these achieving long-term healing [5].

From ancient times up to a few decades ago, treatment had remained unchanged, taking the form of a fistulotomy (“lay-open”) with knife or cautery, or the use of a seton. Despite the high healing rates [6, 7], impaired continence may result from fistulotomy, particularly in patients with high transsphincteric AF [8–10].

In an attempt to achieve the three main treatment goals (i.e., closure of the fistula, preservation of sphincter function, and minimization of healing time [11]), several sphincter-preserving techniques have been described alongside fistulotomy over the last 3 decades. These include endorectal advancement flap (ERAF) [12], biomaterials (fibrin glue [13], fistula plugs [14, 15], adipose-derived stem cells [16]), ligation of the intersphincteric fistula tract (LIFT) [17], video-assisted anal fistula treatment (VAAFT) [18], fistula laser closure (FiLaC™) [19], and the over-the-scope clip (OTSC^®^) Proctology system [20].

While fistulotomy is still regarded by many as the gold standard treatment for low-lying AF, questions remain about how to tailor available surgical options to more complex cases.

Several guidelines are available worldwide for the diagnosis and treatment of AF (cryptoglandular or related to CD) [4, 21–33], but many recommendations are controversial or lack high-quality evidence [34], thus making treatment decisions extremely challenging in the majority of complex cases.

The aim of this study was to explore the contemporary management of cryptoglandular AF, and to obtain a snapshot of the current approaches from surgeons worldwide. Secondary aims were to investigate current surgical options in CD-related AF, and to inform the generation of a surgical treatment algorithm for the various types of AF.

## Materials and methods

All members of the American Society of Colon and Rectal Surgeons (ASCRS) and European Society of Coloproctology (ESCP) were identified through membership directories (ASCRS, *n* = 3126; ESCP, *n* = 415) and were invited by an email to join a fully anonymous online survey (further details provided below). A link to the survey was disseminated via social media (i.e., Twitter and LinkedIn) to capture further potential respondents. A total of four further email reminders (as per software restrictions) were sent throughout the period of online availability of the survey. This study was exempt from review board approval at our institutions.

### Survey

The survey (namely International Survey on Technical Aspects of Anal Fistula Surgery) consisting of a 74-item questionnaire was designed and developed by the first authors (CR & UG) using an online platform [“Online surveys” (formerly BOS—Bristol Online Survey), developed by the University of Bristol]. Co-authors piloted the survey, assessed the design and checked the feasibility and validity of the questions. The finalized online survey was made available online from March 5th to May 28th 2018. The Checklist for Reporting Results of Internet E-Surveys (the CHERRIES statement [35]) is provided as a supplementary file.

The survey aimed to assess crucial elements in surgeons’ evaluation and treatment of perianal abscess and AF, and to capture key demographic information about the respondents. The latter included gender, age group, surgeon’s region of practice and type of hospital (i.e., academic, non-academic teaching, or non-teaching), membership in colorectal societies, regular involvement in research, training level, years of professional experience, and grade of personal experience in AF management during the last year. Other questions assessed the use and usefulness of diagnostic modalities [i.e., endoanal ultrasound (EAUS); magnetic resonance imaging (MRI), computed tomography (CT), and fistulography] in initial workup and guiding treatment strategies for both cryptoglandular and CD-related AF. Incision and drainage of perianal abscess and seton placement were assessed with questions covering preference of site of incision with respect to abscess location, and seton type (i.e., cutting, loose, medicated) and material(s). Surgeons’ views on fistulotomy and fistulectomy were investigated with questions covering the amount of external anal sphincter (EAS) they would safely sacrifice in patients with transsphincteric AF (further delineated with respect to gender), and the possibility of a simultaneous sphincter reconstruction. The use of contemporary sphincter-preserving surgical strategies was also explored, collecting the following data for each technique: number of procedures performed yearly, type(s) of AF in which the technique is considered appropriate, and how the fistula tract is managed. Lastly, participants were asked to declare their rate of recurrence, minor and major fecal incontinence with each technique and separately for cryptoglandular and CD-related AF.

All questions were set as mandatory fields with real-time validation and automated skip logic to prevent missing data and avoid illogical or incompatible responses. Quantitative data were automatically collected by the software and exported to a Microsoft Excel spreadsheet.

### Statistical analysis

Categorical variables across groups were compared using Fisher’s exact test or Pearson’s Chi-square test; 95% confidence intervals (CI) for proportions were calculated using the Wilson method with continuity correction. Binary and ordinal logistic regression models were also fitted to assess the association between surgeons’ preferences and their demographics and experience. The denominator of the percentages of respondents is the total number of colorectal surgeons who took part (*n* = 492). All analyses were performed in STATA 15 (StataCorp LLC, College Station, TX, USA).

## Results

A total of 3708 individuals were approached by email via membership directories of the two above-mentioned colorectal societies (*n* = 3541) and via social media invitation (*n* = 167). There were 3572 (96.3%) appropriate recipients, excluding 136 email addresses listed incorrectly or recognized as spam or with invalid domains.

### Demographics

In total, 510/3572 (14.3%) responded to the survey. Of these, 492 (96%) were surgeons and contributed to the main results (surgical techniques and treatment outcomes). A total of 194 (39%) and 48 (10%) surgeons came from North and South Americas, respectively, 170 (35%) from Europe, 57 (12%) from Asia, 17 (3%) from Australia and New Zealand, and only 2 (0.4%) from Africa, for a total of 66 countries involved (Suppl. Figure 1). Respondents were mostly men (82%), ≥ 40 years of age (64%), colorectal surgeons (84%) at consultant level (84%), practicing in academic (53%) or non-academic teaching (30%) hospitals (Table 1).

**Table 1:** Surgeons’ demographics and experience

	*N* (%)
Geographic distribution	
North America	194 (39)
South America	48 (10)
Europe	170 (35)
Asia	57 (12)
Australia and New Zealand	17 (3)
Africa	2 (< 1)
Gender	
Males	401 (82)
Females	91 (18)
Age group (years)	
< 30	2 (< 1)
30–39	125 (25)
40–49	149 (30)
50–59	129 (26)
≥ 60	87 (18)
Specialty	
Colorectal surgery	414 (84)
General surgery	78 (16)
Training level	
Consultant	412 (84)
Fellow	55 (11)
Resident	25 (5)
Type of hospital	
Academic	260 (53)
Non-academic teaching	147 (30)
Non-teaching	85 (17)
Participate in research^a^	
Local investigator initiated	409 (83)
Multicenter	184 (37)
Industry sponsored	93 (19)
Membership in scientific societies^a^	
ASCRS	304 (62)
No. of Americans (% total American respondents)	228/242 (94)
ESCP	161 (33)
No. of Europeans (% total European respondents)	119/170 (70)
ACPGBI	43 (9)
Others^b^	130 (26)
None	8 (2)
Professional experience with AF management (years)	
> 20	167 (34)
11–20	124 (25)
6–10	124 (25)
0–5	77 (16)
Personal experience in the last year (no. total cases)	
> 50	104 (21)
41–50	45 (9)
31–40	55 (11)
21–30	123 (25)
11–20	117 (24)
1–10	45 (9)
None	3 (1)
Personal experience in the last year (no. CD-related AF)	
> 30	32 (7)
21–30	12 (2)
11–20	67 (14)
6–10	108 (22)
1–5	207 (42)
None	66 (13)

*AF* anal fistula, *CD* Crohn’s disease, *ASCRS* American Society of Colon and Rectal Surgeons, *ESCP* European Society of Coloproctology, *ACPGBI* Association of Coloproctology of Great Britain and Ireland

^a^Multiple answer question

^b^Included 37 further surgical societies worldwide

### Diagnostics in cryptoglandular and CD-related AF

**Fig. 1:**
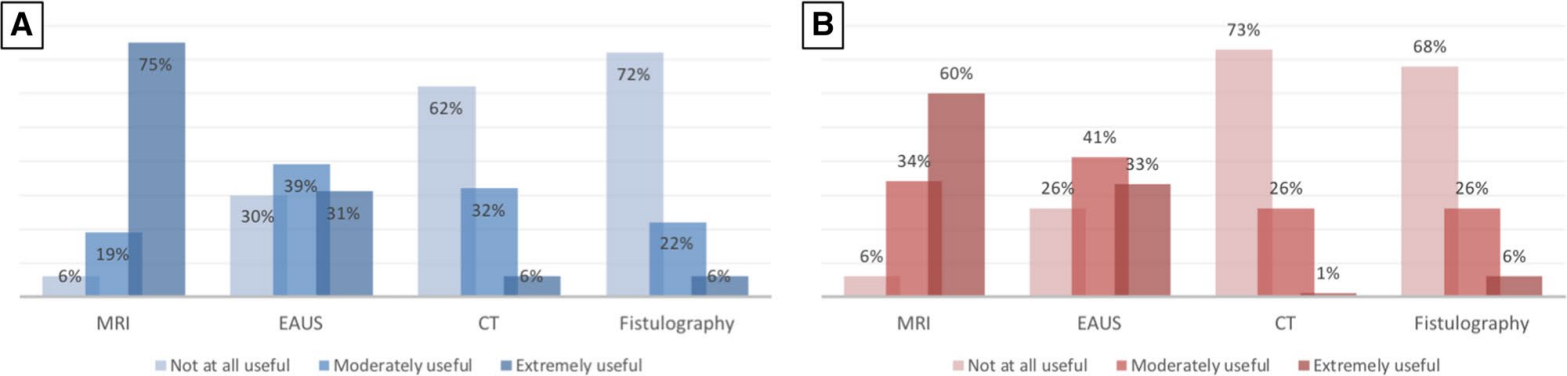
Usefulness ratings of diagnostic modalities in patients with anal fistula (**a** cryptoglandular; **b** Crohn’s disease related). *MRI* magnetic resonance imaging, *EAUS* endoanal ultrasound, *CT* computed tomography

There was strong disagreement among surgeons about the usefulness of endoanal ultrasound (EAUS) in the initial assessment of AF, with only one-third of them finding this test extremely useful in both cryptoglandular and CD-related AF. On the other hand, a significant proportion (nearly 30%) of respondents rated EAUS as not at all useful in any type of fistula (Fig. 1). Americans were much more skeptical than Europeans on the usefulness of EAUS in both cryptoglandular (OR 0.21; *p* < 0.0001) and CD-related AF (OR 0.27; *p* < 0.0001) (Suppl. Table 1). A similar skepticism was evident from surgeons working in non-teaching compared to academic hospitals (Table 2).

**Table 2:** Ordinal logistic regression models to identify the preferred diagnostic modalities for cryptoglandular and Crohn’s disease-related anal fistula (AF) according to surgeons’ demographics and experience

		Diagnostic techniques	Cryptoglandular AF				Crohn’s disease-related AF			
			OR	SE	*p*	95% CI	OR	SE	*p*	95% CI
Geographic distribution	1. Europe (reference)									
	Americas	EAUS	0.207	0.044	**< 0.0001**	0.137–0.315	0.268	0.056	**< 0.0001**	0.178–0.402
		MRI	0.527	0.117	**0.004**	0.341–0.813	0.318	0.087	**< 0.0001**	0.186–0.545
		CT	1.11	0.267	0.658	0.695–1.780	1.13	0.248	0.577	0.735–1.736
		Fistulography	2.25	0.545	**0.001**	1.399–3.615	2.20	0.570	**0.002**	1.329–3.662
	Rest of the world	EAUS	0.619	0.162	0.067	0.370–1.033	0.853	0.222	0.541	0.512–1.421
		MRI	1.134	0.339	0.675	0.631–2.036	0.723	0.270	0.385	0.347–1.504
		CT	0.862	0.283	0.651	0.453–1.641	1.160	0.330	0.601	0.664–2.026
		Fistulography	1.581	0.508	0.154	0.842–2.967	1.839	0.622	0.072	0.947–3.570
Gender	1. Males (reference)									
	Females	EAUS	1.103	0.257	0.673	0.699–1.741	1.458	0.339	0.105	0.925–2.300
		MRI	0.832	0.214	0.475	0.502–1.378	0.768	0.231	0.380	0.427–1.384
		CT	0.938	0.261	0.818	0.543–1.619	1.134	0.281	0.612	0.697–1.843
		Fistulography	1.075	0.285	0.786	0.639–1.807	1.127	0.318	0.671	0.649–1.959
Age group (years)	1. < 40 (reference)									
	40–49	EAUS	0.656	0.185	0.135	0.377–1.140	0.704	0.197	0.211	0.407–1.219
		MRI	1.264	0.411	0.472	0.668–2.391	2.092	0.820	0.060	0.970–4.510
		CT	0.935	0.314	0.841	0.484–1.805	0.587	0.180	0.083	0.322–1.073
		Fistulography	1.009	0.327	0.977	0.535–1.906	0.808	0.286	0.546	0.404–1.615
	50–59	EAUS	0.473	0.197	0.072	0.210–1.069	1.323	0.492	0.452	0.638–2.742
		MRI	1.078	0.489	0.869	0.442–2.625	3.106	1.741	***0.043***	1.035–9.317
		CT	0.562	0.280	0.248	0.212–1.494	0.613	0.265	0.257	0.263–1.429
		Fistulography	0.926	0.953	0.875	0.355–2.418	1.138	0.578	0.800	0.420–3.079
	≥ 60	EAUS	0.444	0.222	0.105	0.166–1.185	0.446	0.213	0.091	0.175–1.137
		MRI	0.760	0.409	0.611	0.265–2.182	1.482	0.942	0.536	0.426–5.152
		CT	0.586	0.347	0.366	0.184–1.869	0.895	0.469	0.832	0.320–2.499
		Fistulography	1.668	0.953	0.371	0.544–5.113	1.437	0.844	0.536	0.455–4.540
Professional experience with AF management (years)	1. > 20 (reference)									
	11–20	EAUS	0.859	0.264	0.620	0.470–1.569	0.998	0.307	0.994	0.546–1.825
		MRI	0.910	0.325	0.792	0.452–1.832	0.228	0.114	***0.003***	0.085–0.608
		CT	0.712	0.255	0.342	0.353–1.435	0.972	0.322	0.933	0.508–1.861
		Fistulography	0.873	0.296	0.689	0.450–1.697	1.220	0.452	0.590	0.591–2.521
	6–10	EAUS	1.319	0.502	0.467	0.626–2.780	1.323	0.492	0.452	0.638––2.742
		MRI	0.517	0.225	0.129	0.220–1.213	0.150	0.089	**0.001**	0.047–0.480
		CT	0.838	0.374	0.692	0.349–2.012	1.551	0.630	0.280	0.699–3.439
		Fistulography	0.647	0.281	0.316	0.276–1.516	0.937	0.449	0.892	0.366–2.398
	0–5	EAUS	1.262	0.630	0.641	0.475–3.356	1.546	0.747	0.367	0.600–3.983
		MRI	0.423	0.235	0.121	0.143–1.254	0.065	0.048	**< 0.0001**	0.015–0.273
		CT	1.349	0.799	0.613	0.423–4.308	1.084	0.574	0.878	0.384–3.060
		Fistulography	1.077	0.617	0.898	0.350–3.313	1.659	0.996	0.399	0.511–5.383
Personal experience in the last year (no. of total cases)	1. < 10 (reference)									
	10–30	EAUS	0.957	0.311	0.893	0.506–1.809	1.166	0.368	0.626	0.629–2.162
		MRI	0.275	0.117	**0.002**	0.119–0.635	0.591	0.294	0.290	0.223–1.565
		CT	0.690	0.243	0.291	0.346–1.375	0.603	0.201	0.129	0.314–1.159
		Fistulography	0.436	0.147	**0.014**	0.225–0.845	0.341	0.118	**0.002**	0.173–0.527
	31–50	EAUS	1.390	0.502	0.362	0.685–2.822	1.430	0.504	0.310	0.717–2.852
		MRI	0.358	0.164	**0.025**	0.146–0.878	0.971	0.528	0.957	0.334–2.820
		CT	0.406	0.168	**0.003**	0.180–0.916	0.536	0.201	0.096	0.257–1.118
		Fistulography	0.443	0.169	**0.033**	0.210–0.936	0.334	0.131	**0.005**	0.155–0.719
	> 50	EAUS	1.385	0.5.03	0.370	0.680–2.821	1.450	0.513	0.294	0.724–2.902
		MRI	0.284	0.131	**0.006**	0.115–0.700	0.625	0.335	0.380	0.219–1.785
		CT	0.484	0.199	0.077	0.216–1.083	0.596	0.223	0.167	0.286–1.242
		Fistulography	0.352	0.140	**0.009**	0.161–0.767	0.235	0.097	**< 0.0001**	0.105–0.527
Training level	1. Consultant (reference)									
	Fellow or resident	EAUS	0.793	0.200	0.359	0.483–1.301	1.268	0.322	0.350	0.770–2.087
		MRI	0.537	0.151	**0.028**	0.309–0.934	0.566	0.180	0.074	0.303–1.057
		CT	0.962	0.288	0.897	0.534–1.731	0.828	0.231	0.498	0.479–1.430
		Fistulography	1.642	0.445	0.067	0.966–2.792	1.401	0.404	0.243	0.796–2.465
Type of hospital	1. Academic (reference)									
	Non-academic teaching	EAUS	0.841	0.169	0.391	0.567–1.249	0.834	0.167	0.364	0.564–1.234
		MRI	0.878	0.192	0.553	0.572–1.350	0.833	0.217	0.483	0.500–1.387
		CT	1.082	0.256	0.737	0.681–1.720	1.341	0.286	0.168	0.883–2.037
		Fistulography	1.360	0.317	0.188	0.861–2.148	1.349	0.327	0.216	0.839–2.169
	Non-teaching	EAUS	0.548	0.134	**0.014**	0.339–0.886	0.548	0.136	**0.015**	0.337–0.892
		MRI	0.927	0.246	0.776	0.551–1.561	0.703	0.209	0.236	0.392–1.259
		CT	0.779	0.238	0.413	0.428–1.417	1.113	0.298	0.690	0.659–1.879
		Fistulography	1.757	0.469	**0.035**	1.041–2.964	1.553	0.436	0.117	0.895–2.694

*OR* odds ratio, *SE* standard error, *CI* confidence interval, *EAUS* endoanal ultrasound, *MRI* magnetic resonance imaging, *CT* computed tomography

Most surgeons rated magnetic resonance imaging (MRI) as extremely useful in the diagnostic workup of cryptoglandular (75%) and CD-related (60%) AF, with only a minority (6%) finding this test ‘not at all useful’. However, compared to Europeans, Americans were less likely to score this test as extremely useful, especially for CD-related AF (OR 0.32; 95% CI 0.19–0.55; *p* < 0.0001; Table 2 and Suppl. Table 1). Compared to those under 40, surgeons between 50 and 59 years of age were more convinced about the usefulness of MRI in CD (OR 3.11; *p* = 0.043), which also positively correlated to the grade of surgeons’ experience, both in terms of number of cases per year and training level (Table 2).

Most surgeons rated computed tomography scanning and fistulography as not at all useful in both cryptoglandular (62% and 72%, respectively) and CD-related (73% and 68%, respectively) AF. Among the 6% of surgeons rating fistulography as extremely useful, most were Americans (Suppl. Table 1). Moreover, this subgroup comprised less experienced surgeons from non-teaching centers.

The main reasons cited by respondents for the preferences in imaging modality were (a) demonstrated higher accuracy over other modalities (74% of respondents); (b) limited personal expertise with other modalities (20%); (c) lack of availability of other modalities (11%); examination under anesthesia (EUA) preferred over diagnostic tests (5%).

Compared to MRI, EAUS was globally less available (96% vs. 76% of surgeons’ working centers, respectively). However, only 56% of surgeons stated that they would personally perform the test. Of these, 62% routinely used three-dimensional (3-D) EAUS and 91% found the injection of hydrogen peroxide into the external orifice helpful during the test.

Among surgeons using MRI preoperatively, the main indications were recurrent (88%) and primary (49%) complex AF, and recurrent simple AF (32%). Similarly, among EAUS users, the main indications were recurrent (37%) and primary (35%) complex AF, and recurrent simple AF (23%).

Only a minority of surgeons stated that they would use MRI (5%) and EAUS (15%) for any type of AF.

More than half (57%) of respondents would arrange an emergency EUA without any preoperative imaging in patients presenting with a perianal abscess, and 18% also in case of primary simple fistula. Similarly, a significant proportion of surgeons would arrange an elective EUA without any preoperative imaging in patients presenting with primary simple fistula (53%) or anal abscess (32%).

### How to drain an abscess

#### Patient position and setting

Overall, 62% of surgeons preferred to drain a perianal abscess with the patient in lithotomy position, followed by prone jack-knife (34%) and Sims (4%) positions. Americans were less likely compared to Europeans to prefer lithotomy position (OR 0.07, CI 0.04–0.13; *p* < 0.0001).

Over half of respondents (59%) would perform incision and drainage both in the operating room (OR) and the outpatient clinic, while 41% would exclusively do this in the OR.

#### Technique (Fig. 2)

The identification of an internal orifice at the time of abscess drainage was more likely deemed ‘necessary’ by older surgeons than by those under 40 years of age (OR 2.28, CI 1.31–3.93; *p* = 0.003).

**Fig. 2:**
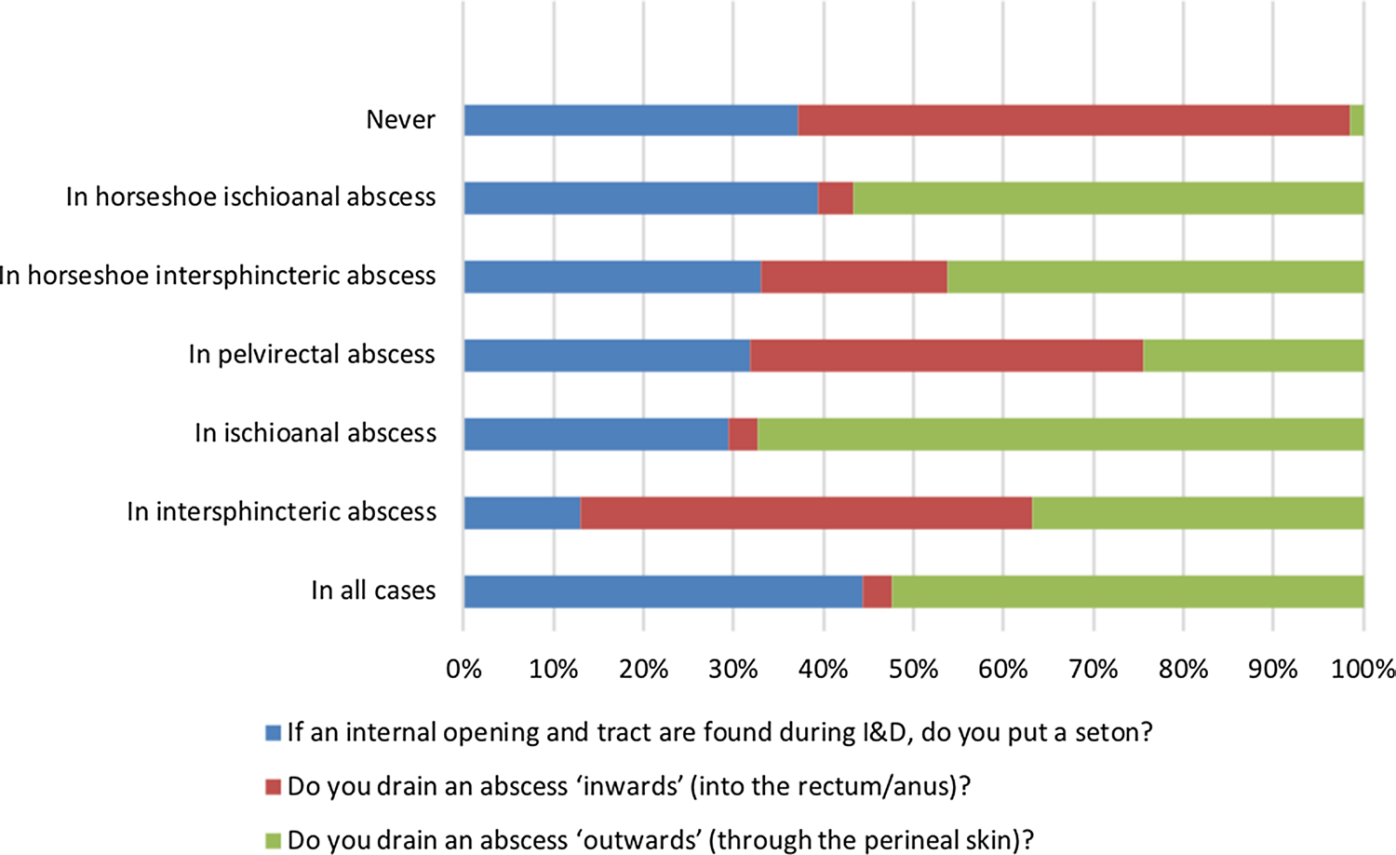
Surgeons’ attitudes towards incision and drainage (I&D) of perianal abscess. NB: multi-answer questions—percentage of respondents who selected each answer option (e.g., 100% would represent that all these question’s respondents chose that option)

Slightly over one in four (28%) surgeons would always place a seton if an internal opening and tract were found during incision and drainage (I&D), while one in six (16%) would never consider this option. A large proportion of respondents would place a seton only in selected cases, depending on the characteristics and location of the abscess [e.g., horseshoe ischioanal abscess (38%)].

When inquired about the preferred site of abscess drainage, most surgeons drained intersphincteric (36%) or pelvirectal (31%) abscesses ‘inwards’ (into the rectum/anus) while 27% would never consider this option. Conversely, over half of the surgeons drained ischioanal or any horseshoe abscess (59%) ‘outwards’ (through the perineal skin), while 27% would consider this option also for intersphincteric abscess, and 33% did so in all cases.

Over a half of the respondents (55%) might consider using a small latex catheter (e.g., Petzer) for I&D of perianal abscess, mainly in patients presenting with deep abscesses (82%).

### Setons

#### Type and material

The majority of respondents reported using loose setons (90%), with fewer utilizing cutting (31%) or medicated (3%) setons. Those using a cutting seton (*n* = 153) reported a median tightening interval of 2 (interquartile range 2–4) weeks. Most surgeons in this group came from the Americas (OR 3.19, CI 1.92–5.31; *p* < 0.0001) and the rest of the world (OR 3.10, CI 1.63–5.88; *p* = 0.001) rather than Europe (Suppl. Table 1). With regard to seton material, vessel loop (silicone) was the most utilized (72%), followed by silk (23%), rubber band (11%), and Prolene (10%) (Suppl. Figure 2). Only six (1%) surgeons stated that they would never use a seton.

#### Scope

Most surgeons (77%) considered the use of setons as part of a staged surgical approach. In this scenario, they would leave the seton in situ for a median of 8 (interquartile range 6–12) weeks to establish a fistula tract before attempting any further definitive treatments. Overall, 70% of surgeons may consider using a seton as a therapeutic strategy in its own right (i.e., palliative loose seton; excl. cutting seton), with over a half (60%) of them considering this option in patients with complex perianal CD.

### Surgical techniques

**Fig. 3:**
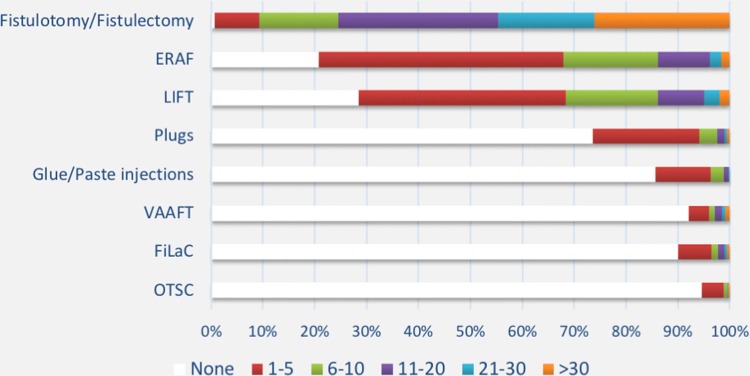
Number of cases per year according to each surgical technique. *ERAF* endorectal advancement flap, *LIFT* ligation of the intersphincteric fistula tract, *VAAFT* video-assisted anal fistula treatment, *FiLaC* fistula laser closure, *OTSC* over-the-scope clip

Surgeons’ experience with the currently available surgical techniques mirrored their chronological development, with the newest approaches (i.e., VAAFT, FiLaC, and OTSC) being performed by less than 10% of the respondents (Fig. 3).

### Fistulotomy and fistulectomy

Fistulotomy and fistulectomy were the most frequently performed operations, with over 40% of respondents carrying out > 20 procedures per year (Fig. 3). Overall, 82% of surgeons considered fistulotomy as the gold standard treatment for simple fistula only, and 13% for most AF. In the latter group, Europeans and surgeons practicing in non-teaching hospitals more commonly used this approach compared to Americans [OR 2.38, 95% confidence interval (CI) 1.18–4.79; *p* = 0.015] and surgeons from academic centers (OR 2.31; 95% CI 1.13–4.49; *p* = 0.021), respectively. During fistulotomy for transsphincteric fistula, in the absence of pre-existing incontinence, surgeons would safely sacrifice up to a median of 25% (interquartile range 16–30%) of the external anal sphincter, with 21% of respondents making no gender distinction. Conversely, a significant proportion (51%) of surgeons would never cut the external sphincter in females with anterior fistula, while another 22% surgeons would never cut at all in female patients.

Less than half of the respondents routinely performed either marsupialization or immediate sphincter reconstruction (primary sphincteroplasty) after fistulotomy for intersphincteric (32% and 9%, respectively) and transsphincteric fistulas (24% and 19%).

One in four surgeons considered fistulectomy an adjunct to sphincter-preserving procedures. Conversely, 15% found this technique a therapeutic strategy in its own right, while another 20% agreed with both uses.

### ERAF

**Fig. 4:**
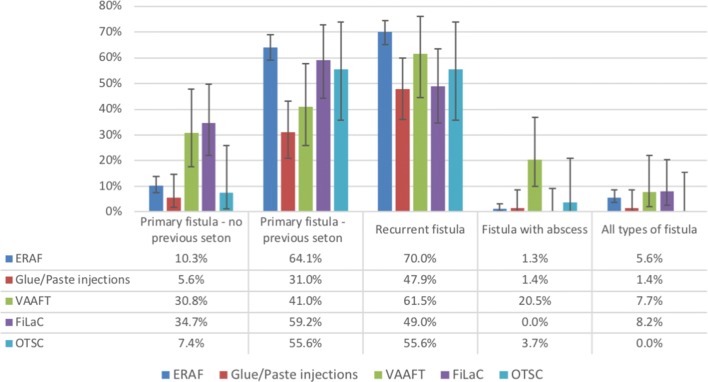
Recommended surgical techniques according to anal fistula type. Bars indicate 95% confidence intervals. *ERAF* endorectal advancement flap, *VAAFT* video-assisted anal fistula treatment, *FiLaC* fistula laser closure, *OTSC* over-the-scope clip

Almost four in five surgeons declared they had some form of experience with ERAF. However, slightly less than 15% of respondents performed > 10 procedures per year (Fig. 3). When asked about the complexity of the operation, more than half (51%) of the respondents found ERAF technically demanding. ERAF was the preferred treatment option for primary AF previously treated with seton (64%) and recurrent AF (70%) (Fig. 4). Seven percent of respondents used ERAF for other indications such as anterior location in females and rectovaginal fistula. Partial (only mucosa–submucosa) or full-thickness (including the internal sphincter) ERAF was equally fashioned (53% and 52% of respondents, respectively), compared to anodermal ERAF, which was performed by only 17% of respondents. In addition to ERAF, curettage was the preferred method of treatment of the fistula tract (65%), followed by fistulectomy (core-out; 43%). Only a minority of surgeons (17%) opted for concomitant additional procedures (e.g., VAAFT; plugs; glue).

### Plugs

Almost one in four surgeons declared experience with plug insertion, with < 3% of respondents overall performing > 10 of these procedures per year (Fig. 3). Surgisis^®^ AFP™ was the most used plug (71%) among the 130/492 surgeons familiar with this technique (Suppl. Figure 3). Before plug insertion, curettage with brush was the preferred method of treatment of the fistula tract (77%), followed by irrigation with saline (39%) or hydrogen peroxide (35%), and curettage with spoon (31%). More than half (59%) of respondents reported placing a seton prior to plug insertion in all cases.

### Glue and paste injection

Less than 15% of surgeons had experience of using glue and paste injection, with only 6/492 (1%) performing > 10 of these procedures per year (Fig. 3). Almost half of the respondents (48%) suggested this treatment option for recurrent AF (Fig. 4). Fibrin glue was the most used infill material (68%) (Suppl. Figure 4). Four in five surgeons usually close the internal opening of the fistula tract after glue/paste injection, using either sutures (84%) or ERAF (36%). Curettage, either with a brush (59%) or spoon (44%), was the preferred method to manage the fistula tract before injection. Compared to those considering glue/paste as a therapeutic strategy in its own right (45%), a higher proportion of surgeons judged this option to be an adjunct to other sphincter-preserving procedures (55%).

### Ligation of the intersphincteric fistula tract (LIFT)

Almost three in four surgeons had experience of performing LIFT. However, only 14% of respondents performed > 10 procedures per year (Fig. 3). When asked about potential contraindications to LIFT, intersphincteric AF (56%) and rectovaginal fistula (50%) were the most common answers. According to 44% of respondents, fistula level was not a determinant of the choice to perform a LIFT, whilst 32% and 19% felt that high and low AF, respectively, may preclude this treatment option.

A significant proportion of surgeons (31%) performed technical variations of the original technique, all listed in Suppl. Figure 5. Of note, fellows or residents were much less likely to add distal fistulectomy compared to surgeons at consultant level (OR 0.21, CI 0.07–0.66; *p* = 0.007).

### VAAFT

Less than 10% of surgeons have used VAAFT, with only 3% overall performing > 10 procedures per year (Fig. 3). The technique was suggested for recurrent AF (62%), AF previously treated with seton (41%) or not (31%) (Fig. 4). One in five surgeons recommended VAAFT also for AF with concomitant abscess. The vast majority of respondents (85%) closed the internal opening after VAAFT. This was generally performed using suture (61%) or ERAF (55%). A minority of surgeons used endostapler (18%) or other devices (e.g., OTSC). The majority of respondents (77%) managed the fistula tract with curettage with brush after VAAFT.

### FiLaC

Overall, 10% of surgeons have used FiLaC, with only 2% of these performing > 10 procedures per year (Fig. 3). FiLaC was the preferred technique to treat AF with no previous seton (Fig. 4). The technique was used as the only treatment option for any type of fistula by 8% of surgeons. Probe withdrawal speed and energy varied among respondents; however, 1 cm per 3 s and 12 W were the most used settings (47% and 38%, respectively). The majority of surgeons (78%) closed the internal opening after FiLaC, with suture (84%).

### OTSC

OTSC was the least used technique, with only 3 (1%) of the surgeons interviewed performing > 10 procedures per year (Fig. 3). The main indications were recurrent AF and AF with previous seton (both 56%).

### Recurrence and incontinence rates

A significant proportion of respondents stated their own recurrence rate was 5% or below after fistulotomy (61%), fistulectomy (29%), and therapeutic seton (29%) for cryptoglandular AF (Suppl. Figure 6). Recurrence rates varied broadly with sphincter-preserving techniques for cyptoglandular AF and with any techniques for CD-related AF. Only 9% and 4% of respondents derived these rates from their own published work in cryptoglandular and CD-related AF, respectively. Almost all respondents (97%) were familiar with seton placement in CD-related AF. Except for fistulotomy (49%), experience with other techniques was very limited (Suppl. Figure 7).

Declared rates of minor and major fecal incontinence were globally low, with better outcomes after sphincter-preserving procedures compared to fistulotomy (Suppl. Figure 8). However, only 4% of surgeons derived such rates from their own published work. Furthermore, less than half of respondents (42%) regularly used validated questionnaires, whilst 11% of them did not routinely assess incontinence preoperatively. Compared to Europeans and Americans, surgeons from the rest of the world were less likely to use validated questionnaires (OR 0.54, CI 0.31–0.96; *p* = 0.036) as well as those practicing in non-academic teaching (OR 0.61, CI 0.40–0.94; *p* = 0.025) and non-teaching hospitals (OR 0.52, CI 0.30–0.88; *p* = 0.016) compared to those from academic centers.

Recurrence and incontinence rates with any surgical techniques did not correlate with surgeons’ experience expressed as number of cases per year or number of years of practice.

## Discussion

### Key results

This is the first study to examine the approach to AF of surgeons worldwide. The survey highlighted lack of consensus regarding the optimal management strategy of patients with AF, demonstrating profound intra- and inter-procedural variations across the various geographic regions and participants’ level of expertise.

### Limitations

This survey provides data from a large number of surgeons worldwide. However, there are a number of limitations worthy of mention. First, the response rate was low (14%). Online surveys do confer several advantages, not least being cost-effective but also reaching people worldwide and allowing prompt and accurate data collection. However, when physicians are asked to take part, they respond poorly and it is common that the response rate is below 20% [36].

Several software restrictions may have negatively affected the response rate. The online surveys email tool did not allow personalized correspondence (known to improve response rate [37]) nor reported bounced emails; if any respondent replied to the email they received from online surveys this was automatically discarded and did not reach survey authors or the support team; a maximum of one invitation email and four reminder emails per respondent (five in total) were sent.

Although we aimed to approach practicing colorectal surgeons (primarily members of two of the most renowned coloproctology societies in the world), the data obtained may not reflect expertise focused on fistula management. Despite the survey being launched worldwide including on social media, it did not reach surgeons, or at least obtain significant response from them in areas such as the Asia-Pacific region and Africa, compared to those in the Americas and Europe, nor did we approach national or reginal coloproctology societies outside of Europe and the Americas. As the questionnaire was designed by surgeons from Europe, the questions may not have adequately represented the views of surgeons from other parts of the world, which may have dissuaded some surgeons from these regions from taking part.

### Interpretation

Management of AF remains challenging, with continuously emerging surgical techniques hampering the development of a robust treatment algorithm. To complicate matters further, the quality of publications is variable in the literature, with many studies having limited follow-up or being too small in size for robust assessment of clinical endpoints [21]. Heterogeneity in outcome measures hampers meta-analysis. Controversies in the published guidelines can be partly explained by the fact that some of them have become outdated because new medical and surgical therapies have been introduced [34]. Moreover, geographic differences also exist.

Among the imaging modalities used to assess AF, our data confirm that most surgeons consider fistulography superseded by other techniques, in line with the second ACPGBI position statement [21], while a minority (mostly Americans) still proclaim its usefulness, in line with the ASCRS Clinical Practice Guidelines [22]. Despite EAUS having an established role in the assessment of AF [21], a significant proportion (nearly 30%) of respondents rated this test as ‘not at all useful’ in any type of fistula. Interestingly, compared to Europeans and those who worked in academic centers, American respondents and surgeons from non-teaching hospitals were much more skeptical of the usefulness of EAUS in the initial assessment of both cryptoglandular and CD-related AF. This may be partly due to limited local availability of EAUS compared to MRI among interviewed surgeons.

There is a proportion of patients who have an internal opening at the time of incision and drainage. Although a randomized clinical trial demonstrated this finding in 83% of patients, AF does not develop in all cases [38]. The identification of an internal orifice at the time of abscess drainage was more likely deemed ‘necessary’ by older surgeons compared to those under 40 years old of age. Sahnan et al. support this finding [39] in selected cases, suggesting that seton placement might be considered in high-risk individuals (e.g., recurrent abscesses) as long as it was performed by an experienced colorectal surgeon, without whom (e.g., out of hours procedure) a simple I&D may suffice.

The vast majority of surgeons (82%) considered fistulotomy the gold standard of treatment for simple fistula. On the other hand, a significant proportion (51%) of surgeons would never cut the external sphincter in females with anterior fistula, while another 22% surgeons would never cut any muscle at all in female patients. This is in line with the ACPGBI recommendation that fistulotomy results in a reliable cure with good patient satisfaction, where 2 cm of proximal muscle remains intact [21].

Our data showed that sphincter-sparing techniques are still in their infancy, with an overall limited level of expertise among interviewed surgeons. About one-third (31%) of respondents performed technical variations of the original LIFT procedure [17], with distal fistulectomy being the most prevalent (Suppl. Figure 5), although this was described in the first description of intersphincteric fistula division in 1993 [40]. LIFT plus distal fistulectomy has been shown to correlate with high healing rates in the mid-term [41] and was more likely performed by surgeons at consultant level compared to fellows or residents.

In conclusion, this survey showed several procedural variations with all the commonly adopted surgical techniques for AF, which are likely to significantly bias the outcomes of published studies and hamper meta-analysis. In addition to patient selection, a meticulous description of all surgical steps should be compulsory for any future scientific contribution on this topic to allow reproducibility and comparison of outcomes among centers. Furthermore, technological innovations should be reported in accordance with the IDEAL collaboration guidelines [42] and application of the core outcome set (COS) should be strongly encouraged to reduce heterogeneity in outcome reporting for CD-related AF [43]. The core outcome set has not been established for cryptoglandular AF yet and it will require a similar rigorous process as the work done for CD-related AF.

## Electronic supplementary material

Below is the link to the electronic supplementary material.

Supplementary material 1 (DOCX 19 kb)

Suppl. Figure 1. Geographic distribution of surgeons (A) with details of country of origin (B). (PNG 1061 kb)

Suppl. Figure 2. Preferred seton materials. Prolene: polypropylene; PTFE: polytetrafluoroethylene; Dacron: polyethylene terephthalate. (PNG 61 kb)

Suppl. Figure 3. Preferred types of plug. (PNG 132 kb)

Suppl. Figure 4. Preferred types of glue and paste. (PNG 167 kb)

Suppl. Figure 5. Technical variations of the Ligation of the Intersphincteric Fistula Tract (LIFT). VALIFT: Video-Assisted LIFT. (PNG 209 kb)

Suppl. Figure 6. Declared recurrence rates after each surgical technique for cryptoglandular (A) and Crohn’s disease-related (B) anal fistulas. ERAF: endorectal advancement flap; LIFT: Ligation of the Intersphincteric Fistula Tract; VAAFT: Video-Assisted Anal Fistula Treatment; FiLaC: Fistula Laser Closure; OTSC: Over-The-Scope Clip. (PNG 878 kb)

Suppl. Figure 7. Experience with fistula surgery in Crohn’s disease. ERAF: endorectal advancement flap; LIFT: Ligation of the Intersphincteric Fistula Tract; VAAFT: Video-Assisted Anal Fistula Treatment; FiLaC: Fistula Laser Closure; OTSC: Over-The-Scope Clip. (PNG 57 kb)

Suppl. Figure 8. Declared rates of minor (A) and major (B) fecal incontinence after each surgical technique for anal fistula. ERAF: endorectal advancement flap; LIFT: Ligation of the Intersphincteric Fistula Tract; VAAFT: Video-Assisted Anal Fistula Treatment; FiLaC: Fistula Laser Closure; OTSC: Over-The-Scope Clip. (PNG 666 kb)

Supplementary material 10 (DOCX 15 kb)
