# Supplementary figures and images for: Contemporary surgical practice in the management of anal fistula: results from an international survey
Source: Tech Coloproctol. 2019 Jul 31;23(8):729–41. doi: 10.1007/s10151-019-02051-5 (PMC6736896; doi:10.1007/s10151-019-02051-5)

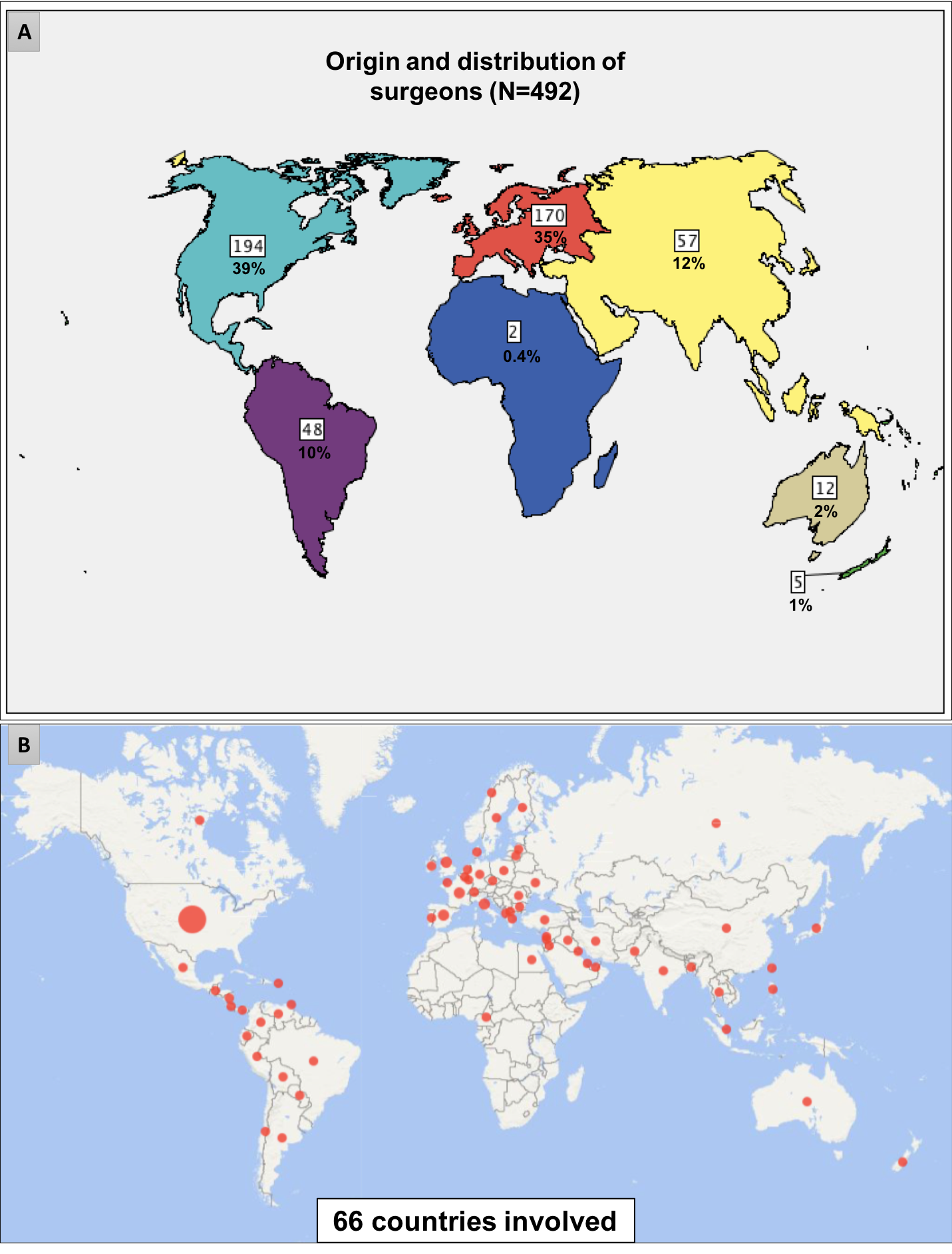

Supplement: Supplementary file 2 — Suppl. Figure 1. Geographic distribution of surgeons (A) with details of country of origin (B). (PNG 1061 kb) [file 10151_2019_2051_MOESM2_ESM.png]

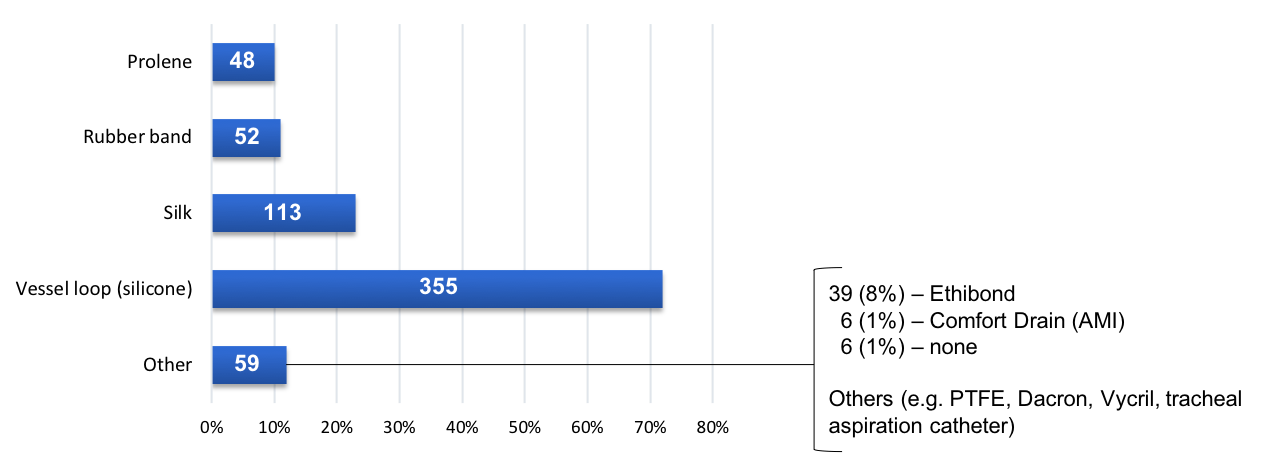

Supplement: Supplementary file 3 — Suppl. Figure 2. Preferred seton materials. Prolene: polypropylene; PTFE: polytetrafluoroethylene; Dacron: polyethylene terephthalate. (PNG 61 kb) [file 10151_2019_2051_MOESM3_ESM.png]

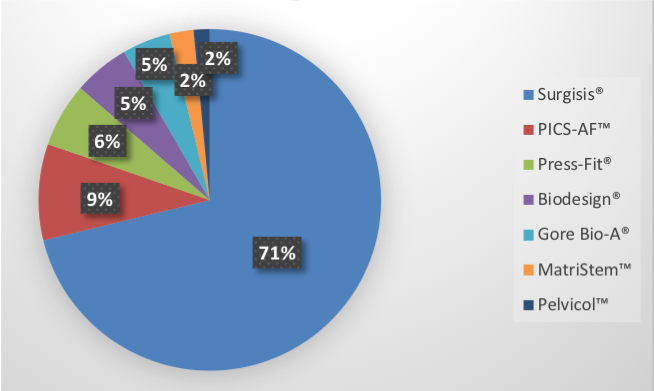

Supplement: Supplementary file 4 — Suppl. Figure 3. Preferred types of plug. (PNG 132 kb) [file 10151_2019_2051_MOESM4_ESM.png]

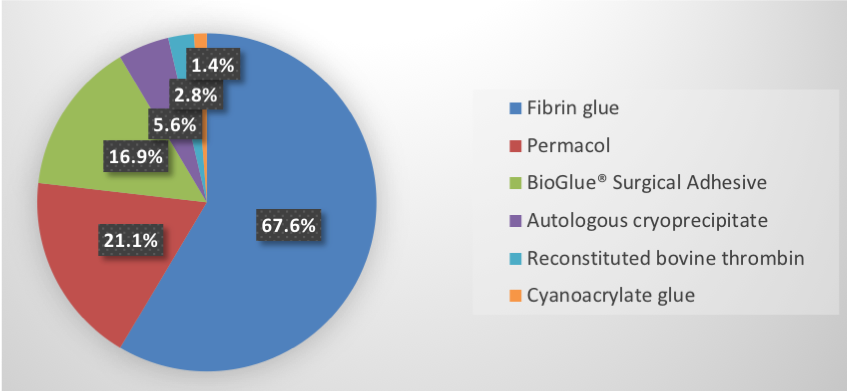

Supplement: Supplementary file 5 — Suppl. Figure 4. Preferred types of glue and paste. (PNG 167 kb) [file 10151_2019_2051_MOESM5_ESM.png]

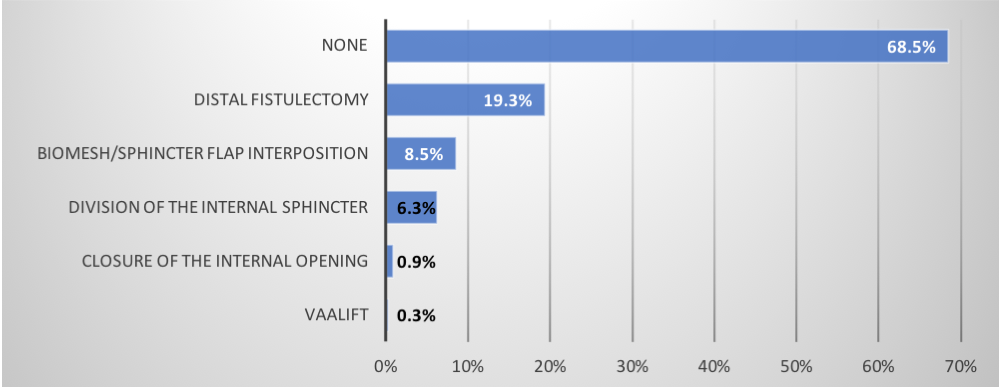

Supplement: Supplementary file 6 — Suppl. Figure 5. Technical variations of the Ligation of the Intersphincteric Fistula Tract (LIFT). VALIFT: Video-Assisted LIFT. (PNG 209 kb) [file 10151_2019_2051_MOESM6_ESM.png]

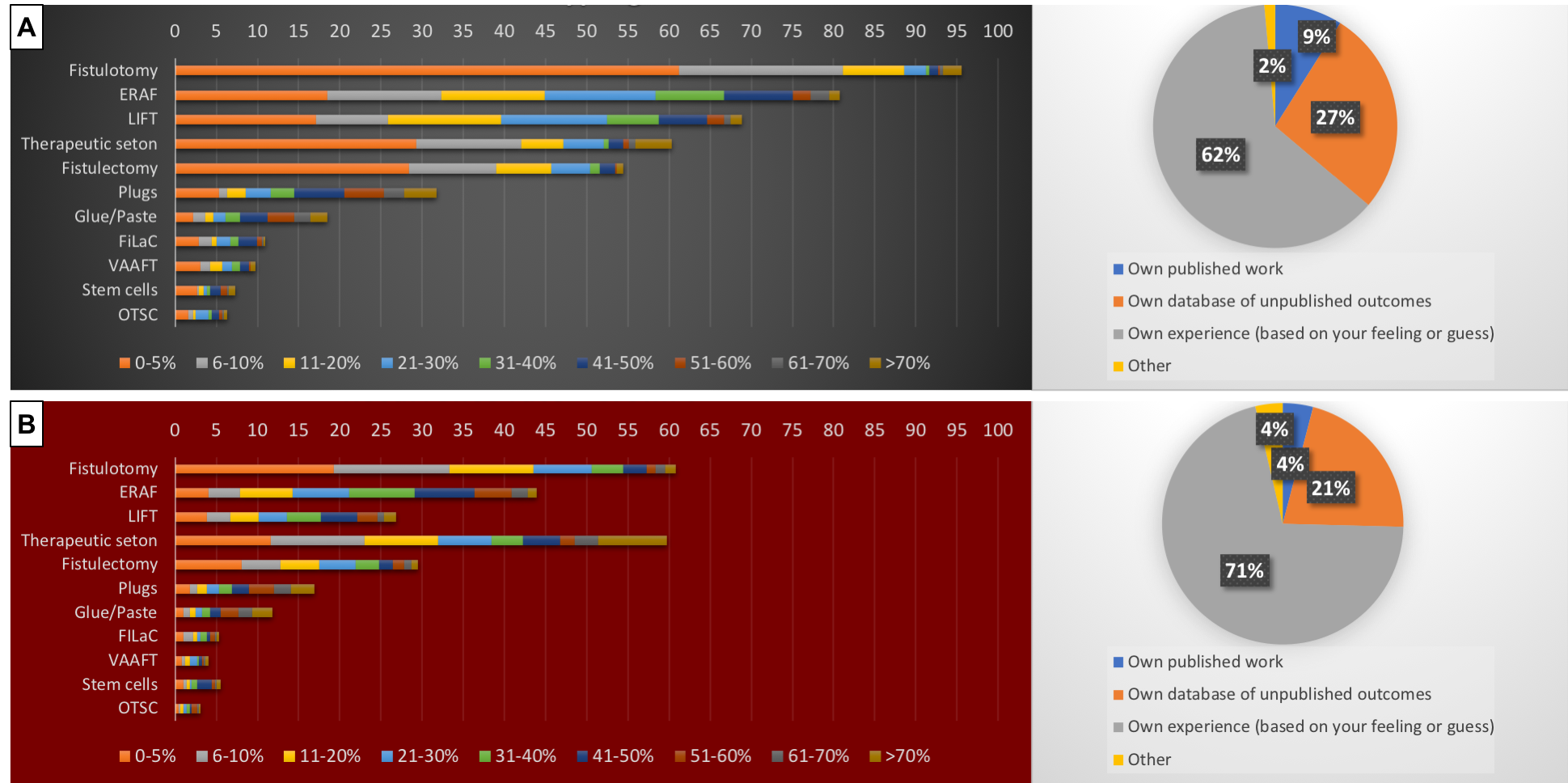

Supplement: Supplementary file 7 — Suppl. Figure 6. Declared recurrence rates after each surgical technique for cryptoglandular (A) and Crohn’s disease-related (B) anal fistulas. ERAF: endorectal advancement flap; LIFT: Ligation of the Intersphincteric Fistula Tract; VAAFT: Video-Assisted Anal Fistula Treatment; FiLaC: Fistula Laser Closure; OTSC: Over-The-Scope Clip. (PNG 878 kb) [file 10151_2019_2051_MOESM7_ESM.png]

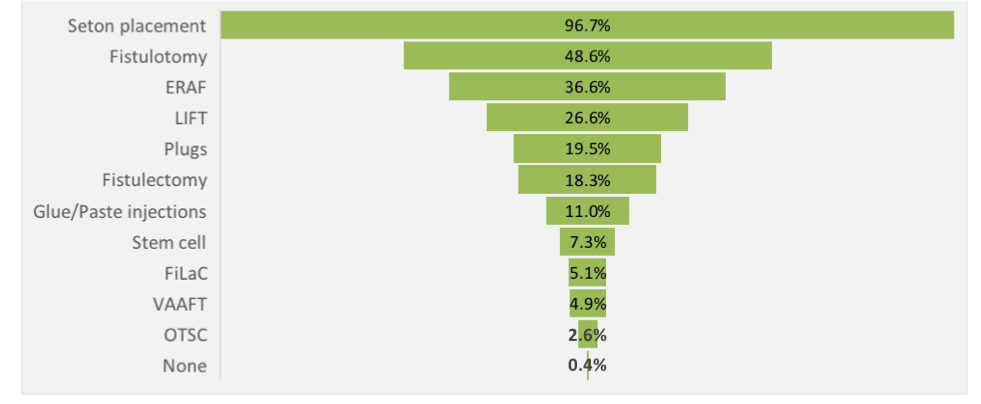

Supplement: Supplementary file 8 — Suppl. Figure 7. Experience with fistula surgery in Crohn’s disease. ERAF: endorectal advancement flap; LIFT: Ligation of the Intersphincteric Fistula Tract; VAAFT: Video-Assisted Anal Fistula Treatment; FiLaC: Fistula Laser Closure; OTSC: Over-The-Scope Clip. (PNG 57 kb) [file 10151_2019_2051_MOESM8_ESM.png]

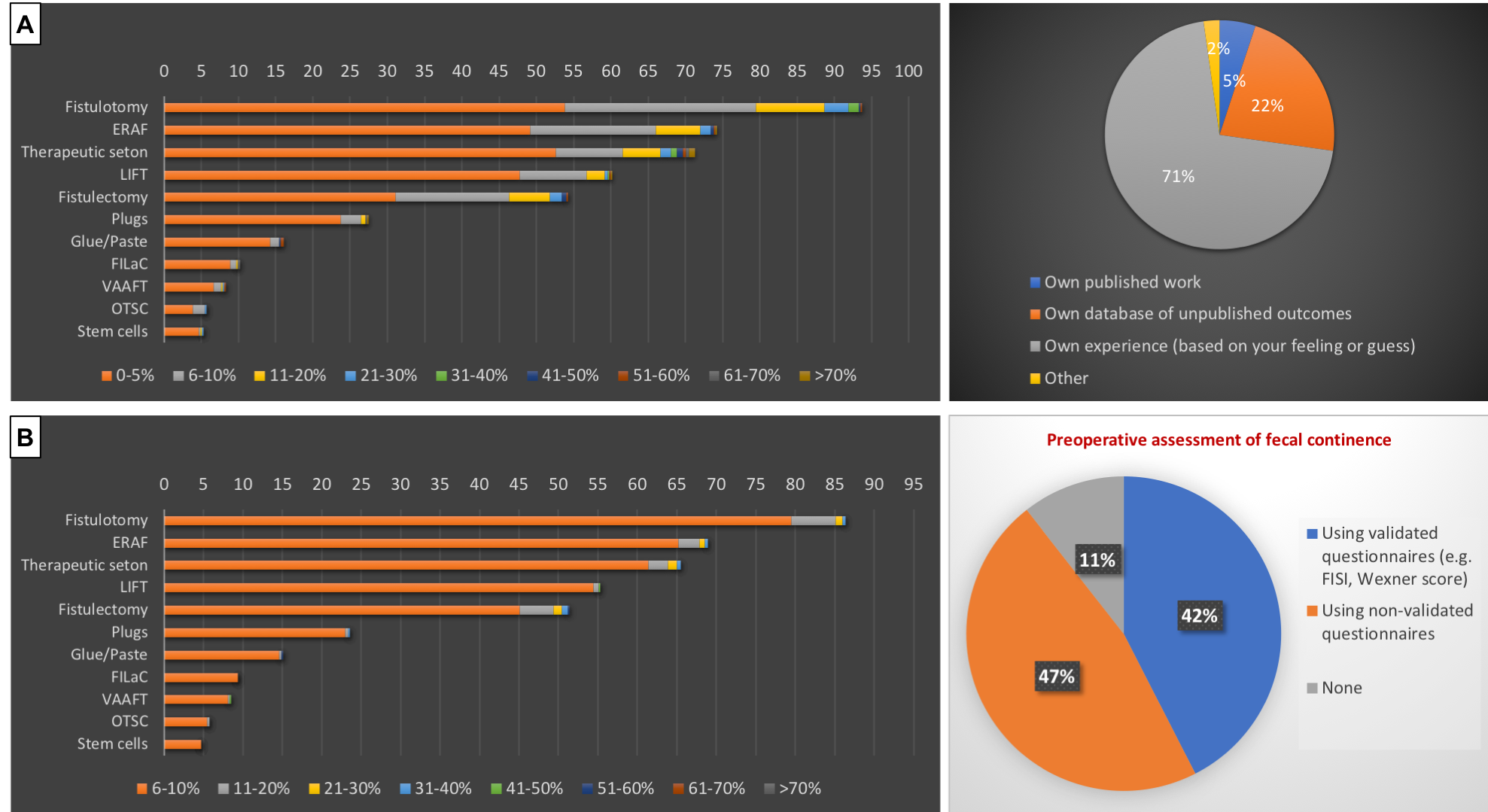

Supplement: Supplementary file 9 — Suppl. Figure 8. Declared rates of minor (A) and major (B) fecal incontinence after each surgical technique for anal fistula. ERAF: endorectal advancement flap; LIFT: Ligation of the Intersphincteric Fistula Tract; VAAFT: Video-Assisted Anal Fistula Treatment; FiLaC: Fistula Laser Closure; OTSC: Over-The-Scope Clip. (PNG 666 kb) [file 10151_2019_2051_MOESM9_ESM.png]
