# Supplementary material for: Contemporary surgical practice in the management of anal fistula: results from an international survey
Source: Tech Coloproctol. 2019 Jul 31;23(8):729–41. doi: 10.1007/s10151-019-02051-5 (PMC6736896; doi:10.1007/s10151-019-02051-5)
Supplement: Supplementary file 10 — Supplementary material 10 (DOCX 15 kb) [file 10151_2019_2051_MOESM10_ESM.docx]

**Suppl. Table 1.** Geographic differences among macro-regions with at least 10% of total respondents.

|  | >20-year experience | Rated as  ‘extremely useful’ (Crypto/Crohn’s) | | | | Use of cutting seton | >30/year fistulotomy or  fistulectomy | >10 procedures/year | | | | | | | >10 Crohn’s AFs |
| --- | --- | --- | --- | --- | --- | --- | --- | --- | --- | --- | --- | --- | --- | --- | --- |
|  |  | E  A  U  S | M  R  I | C  T | F |  |  | E  R  A  F | P  L  U  G  S | L  I  F  T | G  L  U  E | V  A  A  F  T | F  i  L  a  C | O  T  S  C |  |
| Europe (n=170) | 31 | 52/45 | 67/85 | 0/5 | 2/4 | 16 | 27 | 19 | 6 | 10 | 2 | 6 | 5 | 1 | 29 |
| North America (n=194) | 37 | 8/8 | 51/63 | 2/5 | 10/9 | 40 | 22 | 13 | 0 | 12 | 1 | 1 | 0 | 0 | 24 |
| South America (n=48) | 33 | 58/60 | 58/79 | 4/10 | 6/2 | 40 | 21 | 8 | 0 | 13 | 0 | 4 | 0 | 0 | 4 |
| Asia (n=57) | 26 | 35/40 | 70/84 | 0/5 | 4/7 | 32 | 44 | 7 | 2 | 32 | 2 | 0 | 5 | 2 | 19 |

*All values are expressed as percentages. EAUS: endoanal ultrasound; MRI: magnetic resonance imaging; CT: computed tomography; F: fistulography. ERAF: endorectal advancement flap; LIFT: Ligation of the intersphincteric fistula tract; VAAFT: Video-Assisted Anal Fistula Treatment; FiLaC: Fistula Laser Closure; OTSC: Over-The-Scope Clip. AFs: anal fistulas.*
